# Preliminary Prediction of Potential Hepatoprotective Properties of Jujube Extract in Rats Using Metabolomics and Bioinformatics

**DOI:** 10.3390/foods15081407

**Published:** 2026-04-17

**Authors:** Mengyuan Liu, Yali Dang, Shikun Suo, Yanli Wang, Daodong Pan, Xinchang Gao

**Affiliations:** 1 Zhejiang Key Laboratory of Intelligent Food Logistic and Processing, College of Food Science and Engineering, Ningbo University, Ningbo 315211, China; 17513190491@163.com (M.L.); 2301390129@nbu.edu.cn (S.S.); wangyanli@nbu.edu.cn (Y.W.); daodongpan@163.com (D.P.); 2 Institute of Drug Discovery Technology, Ningbo University, Ningbo 315211, China; gaoxinchang@nbu.edu.cn; 3 Qian Xuesen Collaborative Research Center of Astrochemistry and Space Life Sciences, Ningbo University, Ningbo 315211, China

**Keywords:** jujube, metabolomics, absorbed components, network pharmacology, molecular docking, alcoholic liver disease

## Abstract

An integrated approach combining metabolomics, network pharmacology, and molecular docking was employed to systematically explore the serum-absorbed components of jujube, their potential targets, and regulatory pathways. UPLC-MS/MS was used to characterize the absorbed components, while network pharmacology was applied to predict potential targets associated with alcoholic liver disease (ALD). A total of 10 absorbed components and 323 common targets were identified. Among the key components, quercetin, (-)-epigallocatechin, and methyl gallate exhibited strong binding affinities to eight core targets, including AKT serine/threonine kinase 1 (AKT1) and mitogen-activated protein kinase 1 (MAPK1), with quercetin showing the highest content. Jujube intervention significantly altered the serum metabolic profiles of healthy rats, with distinct differences observed between the control and jujube-treated groups. Bioinformatics analysis revealed that the differential metabolites were mainly enriched in the diterpenoid biosynthesis pathway. These findings provide a systematic and preliminary characterization of the serum-absorbed components of jujube, their potential ALD-related targets, and their regulatory effects on serum metabolism in healthy rats. This study provides a preliminary theoretical reference and direction for further research on the potential role of jujube in ALD.

## 1. Introduction

Alcoholic liver disease (ALD) is a chronic liver disorder induced by long-term excessive drinking. It has become a worldwide public health concern. As the core organ for alcohol metabolism, the liver is highly vulnerable to alcohol-related injury. The pathogenesis of ALD involves complex regulatory networks and is affected by multiple factors. Current clinical interventions for ALD mainly rely on abstinence and supportive treatment, and safe and sustainable strategies are still lacking [1]. Moreover, long-term drug intervention tends to cause toxic side effects [2].

Jujube (*Ziziphus jujuba* Mill.) is a widely used medicinal and edible material [3]. It is rich in polyphenols, flavonoids, polysaccharides and other bioactive components, which endow jujube with potential antioxidant and anti-inflammatory activities [4,5]. Existing studies have shown that flavonoids, polysaccharides and polyphenols serve as key bioactive constituents of jujube [6,7,8]. However, the in vivo metabolic characteristics, blood absorption properties, potential molecular targets and pathways of jujube components remain unclear. This is the core scientific issue addressed in the present study. The chemical composition of jujube is complex. Traditional pharmacological studies have mostly focused on overall activity evaluation, lacking systematic component analysis. Therefore, they can hardly illustrate the multi-component and multi-target regulatory characteristics of jujube. Modern analytical techniques and bioinformatic methods enable systematic characterization of absorbed components and their potential molecular mechanisms. Although some studies have suggested that jujube may be potentially related to liver health [9,10], direct functional verification is still insufficient. The blood-absorbed components and their related molecular mechanisms have not been systematically elucidated. Meanwhile, as a natural food, jujube has the advantages of low toxicity, high safety, strong adaptability, and wide distribution [11].

Modern scientific techniques can better illustrate the molecular biological mechanisms of medicinal and edible homologous substances [12]. In this study, based on serum metabolomics, we comprehensively identified the blood-absorbed components of jujube in healthy rats. Combined with network pharmacology and molecular docking [13], we further predicted the potential targets and pathways that may be putatively associated with ALD, providing a theoretical basis for future research. This study clarified the blood-absorbed components of jujube and computationally predicted their potential targets and pathways. The findings will provide a scientific analytical framework and experimental basis for in vivo metabolism research and target exploration of jujube, and will offer theoretical support for the further development of its medicinal and edible value, and related products.

## 2. Materials and Methods

### 2.1. Materials

The dried fruit of *Ziziphus jujuba* Mill. cv. Junzao (Kunyu City, China) was used as the experimental material. Mass spectrometry-grade double-distilled water, methanol, formic acid, and acetonitrile were purchased from Thermo Fisher Scientific Inc., Waltham, MA, USA; quercetin (B20527-100 mg), (-)-epigallocatechin (B20102-20 mg), and methyl gallate (B20853-20 mg) were obtained from Shanghai Yuanye Bio-Technology Co., Ltd., Shanghai, China.

### 2.2. Preparation of Jujube Extract

Jujube (500 g) was mixed with distilled water at a solid-liquid ratio of 1:2 (g/mL) and soaked for 30 min. The mixture was treated with ultrasonication for 40 min at 360 W, followed by high-pressure extraction at 150 kPa and 125 °C for 15 min. Subsequently, the homogenate was centrifuged at 5000 rpm for 15 min, concentrated by rotary evaporation, and freeze-dried to prepare jujube paste aqueous extract (JPAE). The obtained JPAE was reconstituted with distilled water, and the supernatant was collected for subsequent analysis. The resulting supernatant was collected and filtered through a 0.22 μm membrane filter prior to subsequent analysis. All experiments were performed in triplicate. The ultrasonic equipment was purchased from Xinzhi Biotechnology Co., Ltd., Ningbo, China (model SB-400DTY), with an ultrasonic output power of 480 W and an operating frequency of 25 kHz; the pressure cooker was purchased from Shunzhi Food Industry Equipment Co., Ltd., Weifang, China.

### 2.3. Component Analysis and Identification of JPAE

An appropriate amount of sample was reconstituted with water, and an aliquot of the supernatant was collected. The supernatant was diluted with mass spectrometry-grade water to a methanol content of 53% (*v*/*v*). The resulting solution was centrifuged at 15,000× *g* for 20 min at 4 °C. The supernatant was harvested, filtered through a 0.22 μm microporous membrane, and the subsequent filtrate was subjected to UPLC-MS/MS analysis.

### 2.4. Animals and Experimental Design

Twelve male Sprague-Dawley (SD) rats, aged 6–8 weeks with a body weight of 290–320 g, were purchased from Beijing Vital River Laboratory Animal Technology Co., Ltd., Beijing, China. The animal use license number was SYXK (Zhe) 2024-0002, and the certificate number was 20250327Aazz06190270365. The rats were housed in a specific pathogen-free (SPF) environment with constant temperature (21–23 °C) and humidity (50–60%). A 12 h light/dark cycle was applied, and the rats had free access to food and water. After 1 week of acclimatization, the experiment was performed. This study was approved by the Animal Ethics Committee of Ningbo University, and all animal experiments were conducted in accordance with the Guide for the Care and Use of Laboratory Animals (approval number: AEWC-NBU20250336). Twelve SD rats were randomly divided into a control group (*n* = 6) and a JPAE-treated group (*n* = 6). A sample size of *n* = 6 per group was selected based on our research group’s experience with rat models and commonly used sample sizes in relevant published metabolomics studies [14,15]. The rats in the treated group were intragastrically administered with jujube JPAE at a dose of 1.6 g/kg body weight twice daily for 7 consecutive days [16,17]. All administrations were performed between 9:00 a.m. and 5:00 p.m. using a stainless steel gavage needle (16-gauge) attached to a 1 mL syringe.

Rats were fed a standard commercial diet (P1101F-25, SLACOM^®^; Suzhou Shuangshi Laboratory Animal Feed Technology Co., Ltd., Suzhou, China). The diet consisted of fish meal, wheat, corn, soybean meal, wheat bran, vitamins, minerals, and amino acids, with a crude protein content of ≥20.5% and crude fat content of ≥4%, conforming to the national standard GB14924.3-2010.

### 2.5. Serum Sample Preparation

Prior to the last administration, rats were fasted for 12 h. Blood samples (0.5 mL) were collected from the orbital venous plexus of rats at 0, 30, 60, and 120 min, respectively, and centrifuged at 4000 rpm for 10 min at 4 °C. A 100 μL aliquot of serum was transferred into a tube, and 400 μL of 80% methanol aqueous solution was added. The mixture was vortexed and incubated in an ice bath for 5 min, followed by centrifugation at 15,000× *g* for 20 min at 4 °C. An appropriate volume of the supernatant was collected and diluted with mass spectrometry-grade water to a methanol content of 53% (*v*/*v*), then centrifuged again at 15,000× *g* for 20 min at 4 °C. The supernatant was harvested to obtain test substance-containing serum samples, which were subjected to UPLC-MS analysis. Blank serum samples were prepared using the same procedure.

### 2.6. Network Pharmacology Analysis of Blood-Absorbed Components of Jujube

#### 2.6.1. Collection of Potential Targets of Blood-Absorbed Components for ALD Treatment

Target prediction was performed on the identified blood-absorbed components of JPAE by integrating the TCMSP (Traditional Chinese Medicine Systems Pharmacology Database and Analysis Platform) (https://tcmsp-e.com/, accessed on 1 March 2026), SwissTargetPrediction (http://www.swisstargetprediction.ch/, accessed on 1 March 2026) and PharmMapper (http://www.lilab-ecust.cn/pharmmapper/, accessed on 1 March 2026) databases with the PubChem database. The predicted targets and their corresponding genes were standardized using the UniProt database (https://www.uniprot.org) with Homo sapiens set as the species, and valid targets were screened from the potential active components accordingly. With “Alcoholic liver disease” as the keyword, disease-related targets were retrieved from the GeneCards (https://www.genecards.org/, accessed on 1 March 2026), TTD (Therapeutic Target Database) (http://db.idrblab.net/ttd/, accessed on 1 March 2026) and OMIM (Online Mendelian Inheritance in Man) (https://www.omim.org/, accessed on 1 March 2026) databases. After merging the retrieved data and removing duplicate entries from the three databases, the ALD-associated targets were obtained.

#### 2.6.2. Construction of Protein-Protein Interaction (PPI) Network and Screening of Core Targets

The PPI network was constructed using the Search Tool for the Retrieval of Interacting Genes/Proteins (STRING, https://cn.string-db.org). The data were imported into Cytoscape 3.10.3 software, and the degree value was applied as the criterion for screening key targets.

#### 2.6.3. GO and KEGG Pathway Analysis

Gene Ontology (GO) and Kyoto Encyclopedia of Genes and Genomes (KEGG) enrichment analyses were performed using the DAVID database (https://davidbioinformatics.nih.gov, accessed on 2 March 2026). Genes were mapped to the nodes of the Gene Ontology database, and enrichment analysis was conducted via the Gene Ontology resource (http://www.geneontology.org/, accessed on 2 March 2026). The target proteins classified into biological process GO_BP, GO_CC, and GO_MF were presented in three independent manners. KEGG pathway enrichment analysis for the intersecting targets was carried out using the KEGG pathway database (www.kegg.jp/kegg/pathway.html, accessed on 2 March 2026) with the criterion of *p* < 0.05.

### 2.7. Molecular Docking

With the blood-absorbed components as ligand molecules and the screened core targets as receptors, molecular docking verification was performed. The chemical structures of the ligands were obtained from the PubChem database (https://pubchem.ncbi.nlm.nih.gov/, accessed on 2 March 2026) and exported in SDF format. The protein structures in PDB format were retrieved from the RCSB Protein Data Bank (https://www.rcsb.org/, accessed on 2 March 2026).

The structures of putative bioactive components were imported into Discovery Studio 2019 (Dassault Systèmes, San Diego, CA, USA), and the Ligand Preparation function was used to complete ligand processing. For putative target proteins, the “Clean Protein” function in the “Prepare Protein” section on the Macromolecules page was used to complete receptor protein processing. CDOCKER semi-flexible docking (Discovery Studio 2019) was performed to investigate the binding interactions between putative bioactive components and putative target proteins. The binding site was defined around the active center of each putative target, and standard docking protocols were applied. The compound-target docking simulation was carried out with the -CDOCKER option to analyze the binding interactions between ligands and proteins [18].

### 2.8. Serum Metabolomic Analysis of JPAE

#### 2.8.1. UPLC-MS/MS Analysis Conditions

Chromatographic separation was performed on a Hypersil Gold C18 column (100 mm × 2.1 mm, 1.9 μm, Thermo Fisher Scientific, Waltham, MA, USA). Liquid chromatography analysis was carried out using a Vanquish UPLC system (Thermo Fisher Scientific, Waltham, MA, USA). Mobile phase A was 0.1% formic acid, and mobile phase B was methanol. The injection volume was 5 μL. The gradient elution program was set as follows: 0 min: 2% methanol, 1.5 min: 2% methanol, 3 min: 85% methanol, 10 min: 100% methanol, 10.1 min: 2% methanol, 11–12 min: 2% methanol. The flow rate was 0.20 mL/min, and the column temperature was 40 °C.

Mass spectrometry detection was performed using a Q-Exactive mass spectrometer (Thermo Fisher Scientific, Waltham, MA, USA). The full-scan mass range was set at m/z 100–1500. The parameters for the electrospray ionization (ESI) source were set as follows: spray voltage: 3.5 kV; sheath gas flow rate: 35 psi; auxiliary gas flow rate: 10 L/min; capillary temperature: 320 °C; S-lens RF level: 60; auxiliary gas heater temperature: 350 °C. Data were acquired in both positive and negative ion modes over a scan range of m/z 250–1000. The MS/MS scan was operated in data-dependent acquisition (DDA) mode.

#### 2.8.2. Qualitative and Quantitative Detection

The raw data files were preprocessed using XCMS software (version 3.13; The Scripps Research Institute, La Jolla, CA, USA), and the parameters (e.g., retention time and mass-to-charge ratio) of each metabolite were screened. First, peak extraction, peak quantification and peak alignment were performed, followed by matching with the high-quality MS/MS spectral database according to the set parameters including ppm and adduct ions. Subsequently, the peak area was calibrated with the first quality control (QC) sample to improve the identification accuracy. Peak extraction was then conducted with the set criteria of a mass deviation of 5 ppm, a signal intensity deviation of 30%, minimum signal intensity and adduct ions, and the peak areas were simultaneously quantified. Target ions were further integrated, after which the molecular formulas were predicted based on molecular ions and fragment ions and matched with the mzCloud (https://www.mzcloud.org/, accessed on 2 February 2026), mzVault (Thermo Fisher Scientific, Waltham, MA, USA, version 4.0), MSDIAL (version 5.5.250530, Kyoto University, Kyoto, Japan) and Masslist databases. Compound information was confirmed by analyzing MS2 fragment ion data, comparing with standard reference materials and relevant literature. Compounds with no matching results were removed, and only those with clear molecular formula and structural information were retained. Furthermore, to evaluate the quality and robustness of the OPLS-DA models and to exclude the possibility of overfitting, permutation tests (*n* = 200) were performed. In each permutation, the class labels of samples were randomly reassigned, followed by model recalculation. The resulting R^2^ and Q^2^ values from the permuted models were compared to those of the original model.

### 2.9. Quantification of Putative Bioactive Target Components from JPAE

LC-MS System Information: LC-MS analysis was performed on a SHIMADZU LC-40C system coupled with a SCIEX Triple Quad™ 4500 mass spectrometer. Chromatographic separation was conducted on a Phenomenex^®^ Kinete 2.6 μm Polar C18 100 Å column (2.1 mm × 30 mm, Phenomenex Inc., Torrance, CA, USA). Mobile phase A was 0.1% formic acid in water, and mobile phase B was 100% acetonitrile. The gradient elution program was set as follows: 0 min: 2% acetonitrile, 0.5 min: 2% acetonitrile, 2.5 min: 25% acetonitrile, 3.5 min: 90% acetonitrile, 4.5 min: 90% acetonitrile, 4.6 min: 2% acetonitrile, 6 min: 2% acetonitrile. The flow rate was set at 0.30 mL/min. The analytical method was validated for linearity, precision, and repeatability. Linearity was evaluated using external standard curves. Precision was assessed by repeated injections of standard solutions, and repeatability was evaluated by replicate sample preparation and analysis. MRM Mass Spectrometry Parameters are shown in Table 1.

### 2.10. Statistical Analysis

Statistical analysis was performed using GraphPad Prism version 10.1.2 (GraphPad Software, San Diego, CA, USA) and the metabolomic data processing software metaX (version 1.1.6, BGI-Shenzhen, Shenzhen, China). For univariate statistical analysis, significance was analyzed using analysis of variance (ANOVA) or Student’s *t*-test, and the results were expressed as mean ± standard deviation (SD). A *p* value < 0.05 was considered statistically significant. GraphPad Prism version 10.1.2 was used for the statistical analysis of quantitative component histograms and molecular docking heatmaps.

For multivariate statistical analysis of metabolomic data, after data transformation using metaX, principal component analysis (PCA) and orthogonal partial least squares discriminant analysis (OPLS-DA) were performed to visualize the separation between groups and obtain the variable importance in projection (VIP) values of each metabolite. The validity and robustness of the OPLS-DA model were further verified by permutation tests with 200 iterations. In the univariate analysis part, Student’s *t*-test was used to calculate the statistical significance (*p*-value) of each metabolite between groups, and the fold change (FC) value of metabolites between groups was calculated. Volcano plots were generated to visualize significantly altered metabolites. The default criteria for screening differential metabolites were VIP > 1, *p* < 0.05, and FC ≥ 2 or FC ≥ 0.5. Given the exploratory nature of untargeted metabolomics, false discovery rate (FDR) control was not applied in the present study. All interpretations of pathway and functional enrichment were treated as putative and hypothesis-generating, and further validation with targeted metabolomics will be performed in future studies to confirm the differential metabolites and related statistical significance.

## 3. Results and Discussion

### 3.1. Analysis of Blood-Absorbed Components of JPAE

During the 7-day administration period, no mortality or abnormal behavioral changes were observed in any group. Rats in the JPAE-treated group exhibited normal activity, feeding, and drinking behavior comparable to those in the control group.

**Figure 1:**
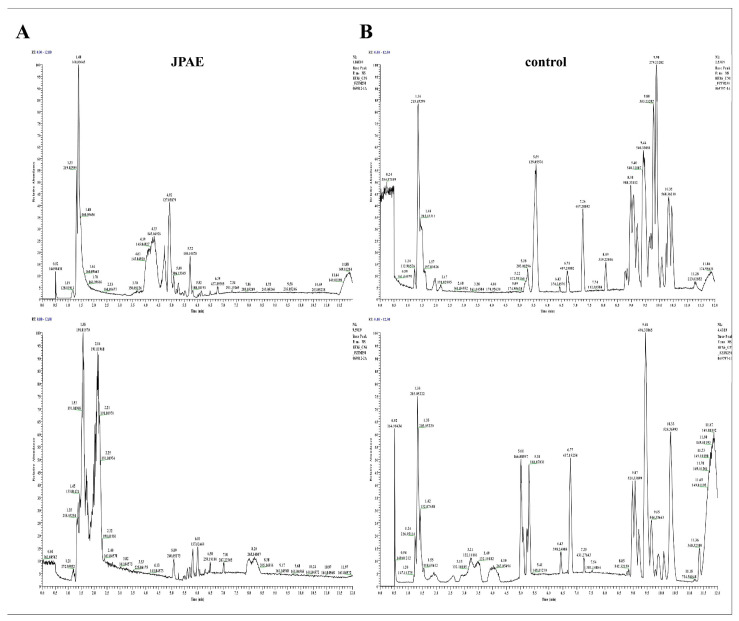
BPCs of plasma samples analyzed by UPLC-MS in positive and negative ion modes. (**A**) JPAE group, positive ion mode and negative ion mode; (**B**) rat serum samples from the control group, positive ion mode and negative ion mode.

**Figure 2:**
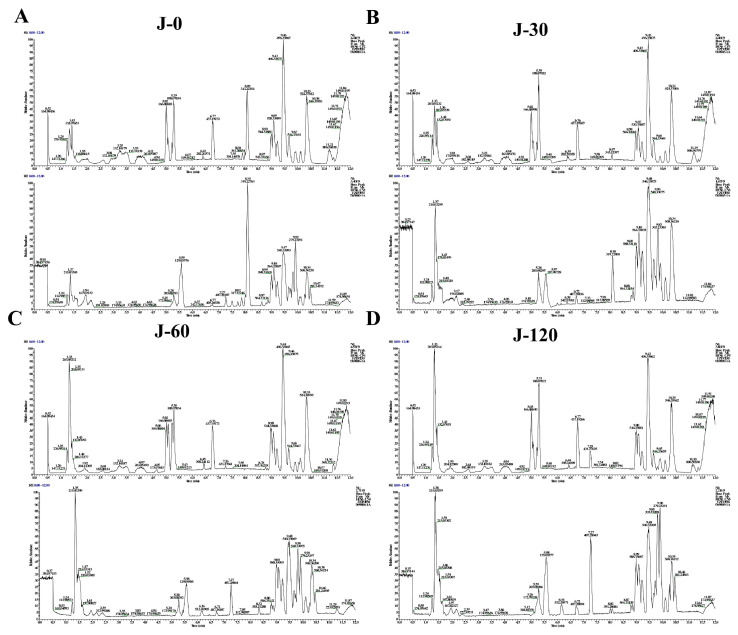
BPCs of rat serum samples at different time points after oral administration of JPAE, analyzed by UPLC-MS in positive and negative ion modes. (**A**) J-0 group (0 min), positive ion mode (upper panel) and negative ion mode (lower panel); (**B**) J-30 group (30 min), positive ion mode (upper panel) and negative ion mode (lower panel); (**C**) J-60 group (60 min), positive ion mode (upper panel) and negative ion mode (lower panel); (**D**) J-120 group (120 min), positive ion mode (upper panel) and negative ion mode (lower panel).

Figure 1A show the BPCs (base peak chromatogram) of JPAE samples in both ion modes. Figure 1B and Figure 2 present the BPCs of serum from rats in the control group and the group treated with the test substance, respectively. These BPCs were obtained in negative and positive ion modes, respectively. Peaks were identified by comparing with databases and sample data, according to retention time and mass spectral fragments. We compared the BPCs of the test substance, treated rat serum, and blank serum. Compounds found in both the test substance and treated rat serum were recognized as prototype blood-absorbed components. These compounds showed no obvious response in blank serum. Compounds detected only in treated rat serum were identified as differential metabolites. They had no apparent response in the test substance or blank serum. We acknowledge that the rat food was not analyzed for the presence of jujube-derived compounds. However, the standard commercial chow used in this study does not contain jujube, and the control group received vehicle only, indicating that the observed effects can be attributed to the JPAE intervention. Future studies may benefit from analyzing both the intervention and diet to further exclude potential confounding factors. Based on mass spectral analysis and NovoMagic database matching, a total of 10 candidate absorbed compounds from JPAE were identified. Candidate absorbed compounds in rat serum were identified based on accurate mass and MS/MS fragmentation spectra. All compounds were putatively annotated by matching against a local in-house database (Novogene), with a mass error threshold of <5 ppm. These components included 3 flavonoids, 6 phenolic acids, and 1 glycoside. The identified components are listed in Table 2.

### 3.2. Network Pharmacology Analysis Based on Blood-Absorbed Components

#### 3.2.1. Prediction of Potential Targets for Blood-Absorbed Components

**Figure 3:**
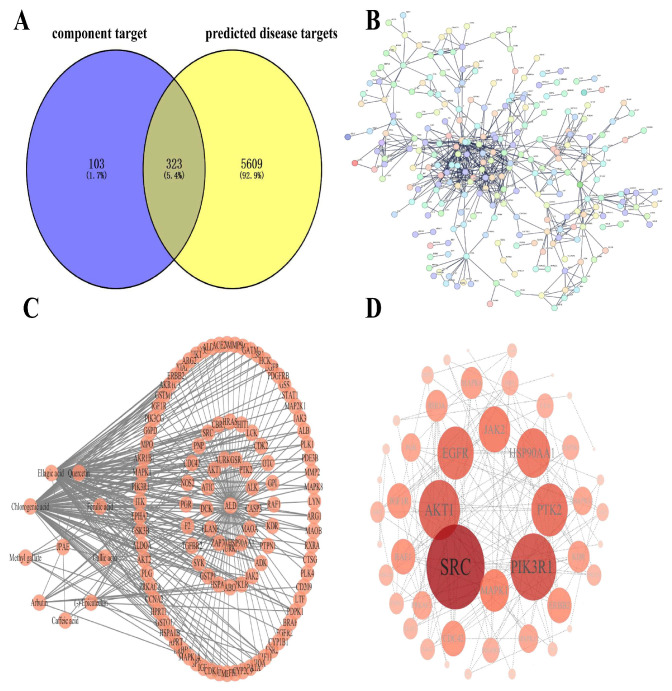
Network pharmacology analysis of JPAE putative components anti-ALD. (**A**) Venn diagram of overlapping targets between JPAE components and ALD-related targets; (**B**) PPI network of overlapping targets; (**C**) Component-target network of jujube putative components and their corresponding targets; (**D**) Putative targets interaction network (node size represents degree value).

After database screening and deduplication, 423 component targets were obtained for the candidate absorbed compounds of JPAE. Using “alcoholic liver disease” as the keyword, disease targets were retrieved from the GeneCards, TTD, and OMIM databases. After merging and deduplication, 5609 targets associated with ALD were obtained. Among them, targets with a relevance score ≥ 10 for alcoholic liver injury were screened from the GeneCards database. As shown in Figure 3, 323 intersection targets between component-related targets and ALD-related targets were identified using a Venn diagram. The constructed network contained 322 nodes and 617 edges, with an average degree value of 3.83. Nodes represented target proteins, and edges represented the interactions between proteins.

#### 3.2.2. Functional Enrichment and Pathway Analysis of Targets

**Figure 4:**
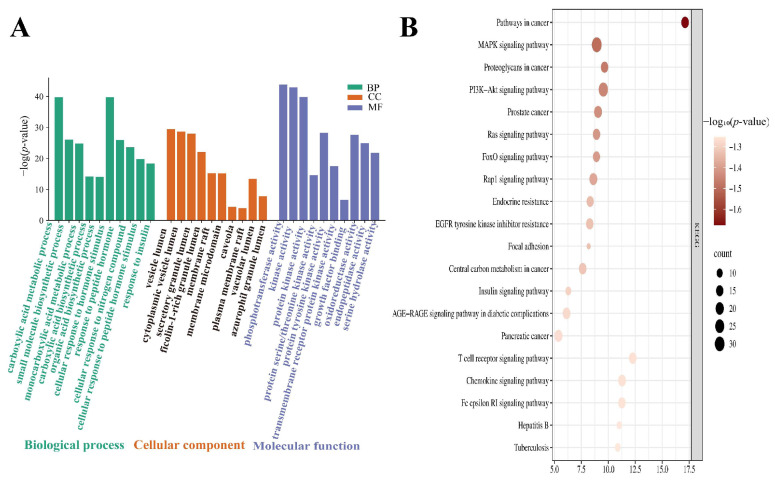
Functional enrichment analysis. (**A**) GO enrichment analysis, including biological processes (BP, green), cellular components (CC, orange), and molecular functions (MF, blue); (**B**) KEGG pathway enrichment analysis.

To explore the potential association between the candidate absorbed compounds of JPAE and ALD, we performed GO functional enrichment analysis on the intersecting targets (Figure 4A). The results showed that these targets were significantly enriched in biological processes including carboxylic acid metabolic process, small molecule metabolic process, organic acid metabolic process, and cellular response to hormone stimulus. These observations suggest that the candidate absorbed compounds of JPAE may be involved in processes related to metabolic homeostasis and hormone response, which may be potentially associated with alcohol-induced metabolic disorders.

With respect to cellular components, the targets were mainly distributed in vesicles, cytoplasm, membrane rafts, and other structures, indicating that their putative sites of action may be concentrated in regions associated with membrane transport and intracellular signal transduction.

At the molecular function level, the targets were highly enriched in protein kinase activity and transmembrane receptor protein kinase activity, which are key nodes in the regulation of inflammation and cellular signaling. These findings imply that the candidate absorbed compounds of JPAE may be associated with kinase-related activities and may participate in related regulatory processes.

Furthermore, KEGG pathway enrichment analysis was performed (Figure 4B) to explore putative signaling pathways associated with the candidate absorbed compounds of jujube. The results showed that the intersecting targets were mainly enriched in pathways including the MAPK signaling pathway, PI3K-Akt signaling pathway, insulin resistance pathway, and AGE-RAGE signaling pathway [19,20]. These pathways have been widely reported to be related to inflammatory response, oxidative stress, apoptosis, and metabolic disorders in the literature [21].

Based on the bioinformatic inference, these enriched pathways may be related to processes associated with inflammation and oxidative stress. In addition, these pathways have been reported to be involved in the pathogenesis and progression of ALD. However, all the above results were obtained from bioinformatic prediction and enrichment analysis. Further experimental verification is necessary to elucidate the precise mechanism of action.

### 3.3. Molecular Docking

**Figure 5:**
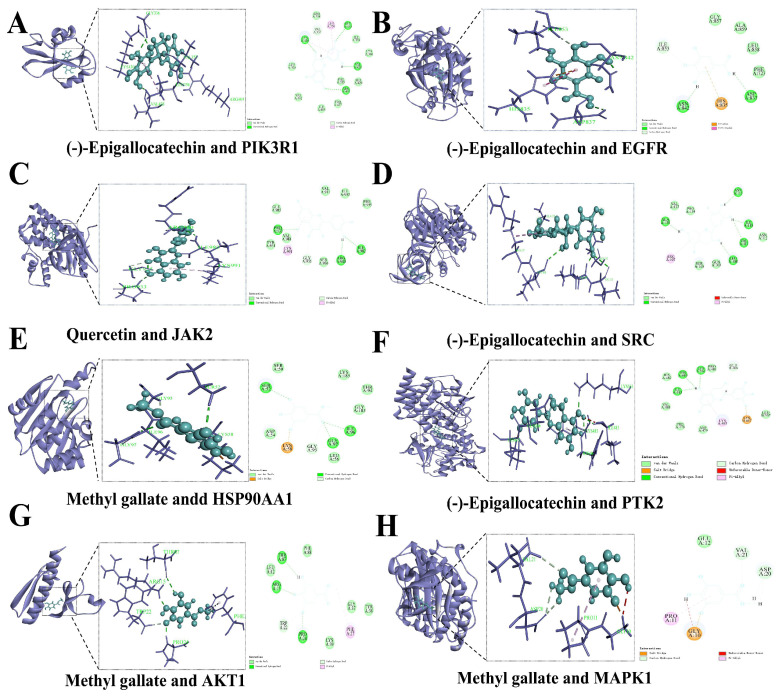
Molecular docking analysis of putative absorbed components from JPAE with targets. (**A**) Docking of (-)-Epigallocatechin with PIK3R1; (**B**) Docking of (-)-Epigallocatechin with EGFR; (**C**) Docking of (-)-Epigallocatechin with SRC; (**D**) Docking of (-)-Epigallocatechin with PTK2; (**E**) Docking of Quercetin with JAK2; (**F**) Docking of Methyl gallate with HSP90AA1; (**G**) Docking of Methyl gallate with AKT1; (**H**) Docking of Methyl gallate with MAPK1.

Molecular docking simulation was performed between the candidate absorbed components of JPAE and putative ALD-related targets (SRC, AKT1, PIK3R1, JAK2, HSP90AA1, MAPK1, PTK2, EGFR). As shown in Figure 5, methyl gallate, (-)-epigallocatechin, and quercetin exhibited relatively low binding energies with these targets [22]. In addition, targets such as SRC, MAPK1, and AKT1 showed favorable predicted binding affinity with various components [23,24], implying that these might represent potential candidate targets of JPAE.

**Figure 6:**
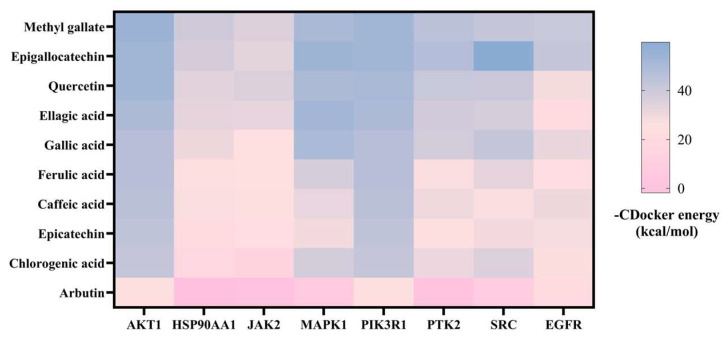
Heatmap of -CDocker energy between 10 active components of JPAE and 8 putative targets.

Molecular docking results (Figure 6) further showed that methyl gallate could bind to HSP90AA1, PIK3R1, AKT1, and MAPK1 via hydrogen bonds and hydrophobic interactions, and might occupy their active pockets [25]. These computational findings suggested that putative bioactive components of JPAE could interact with putative targets, offering a basis for further exploration of their possible relationships with inflammation-related and oxidative stress-related processes.

This study developed a multi-dimensional approach combining serum metabolite profiles, putative bioactive components, and putative targets. This framework aided in characterizing circulating components and provided foundational support for investigating the observed links between putative bioactive components of JPAE and their biological activities.

### 3.4. Determination of Putative Bioactive Components in JPAE

**Figure 7:**
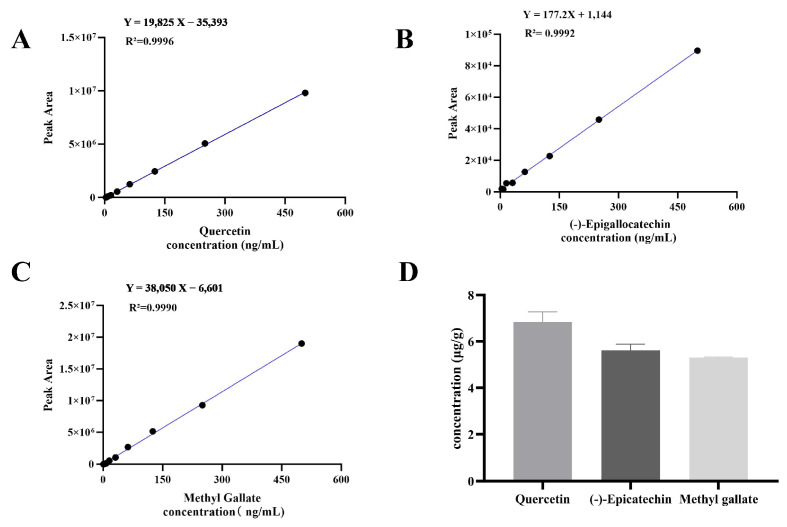
Quantitative analysis of putative components in JPAE (**A**) Standard curve of quercetin (Y = 19,825X − 35,393, R^2^ = 0.9996); (**B**) Standard curve of (-)-epigallocatechin (Y = 177.2X + 1144, R^2^ = 0.9992); (**C**) Standard curve of methyl gallate (Y = 38,050X − 6601, R^2^ = 0.9990); (**D**) Concentrations of quercetin, (-)-epigallocatechin, and methyl gallate in JPAE (μg/g). (Data are expressed as mean ± SD (*n* = 3)).

Standard curves were established with peak area (Y) versus analyte concentration (X) (Figure 7A–C). For quercetin, the regression equation was Y = 19,825X − 35,393 (R^2^ = 0.9996), with precision and repeatability RSD of 0.56% and 1.1%, respectively. It showed good linearity in the range of 0–500 ng/mL. For (-)-epigallocatechin, the regression equation was Y = 177.2X + 1144 (R^2^ = 0.9992), with precision and repeatability RSD of 0.65% and 1.4%, respectively. It showed good linearity in the range of 0–500 ng/mL. For methyl gallate, the regression equation was Y = 38,050X − 6601 (R^2^ = 0.9990), with precision and repeatability RSD of 0.76% and 1.7%, respectively. It showed good linearity in the range of 0–500 ng/mL. All results indicated that the method was reliable and suitable for quantitative analysis. Based on LC-MS detection, the contents of target components in JPAE were determined to be 6.83 ± 0.44 μg/g for quercetin, 5.62 ± 0.26 μg/g for (-)-epigallocatechin, and 5.31 ± 0.03 μg/g for methyl gallate (mean ± SD, n = 3, Figure 7D).

Although these putative bioactive components showed low contents in the extract, they exhibited strong binding with putative targets. It is suggested that these candidate absorbed compounds may be involved in the regulation of related signaling pathways, which may be related to the overall regulatory properties of JPAE.

### 3.5. Serum Metabonomics Analysis

#### 3.5.1. Principal Component Analysis of Serum Metabolomics

**Figure 8:**
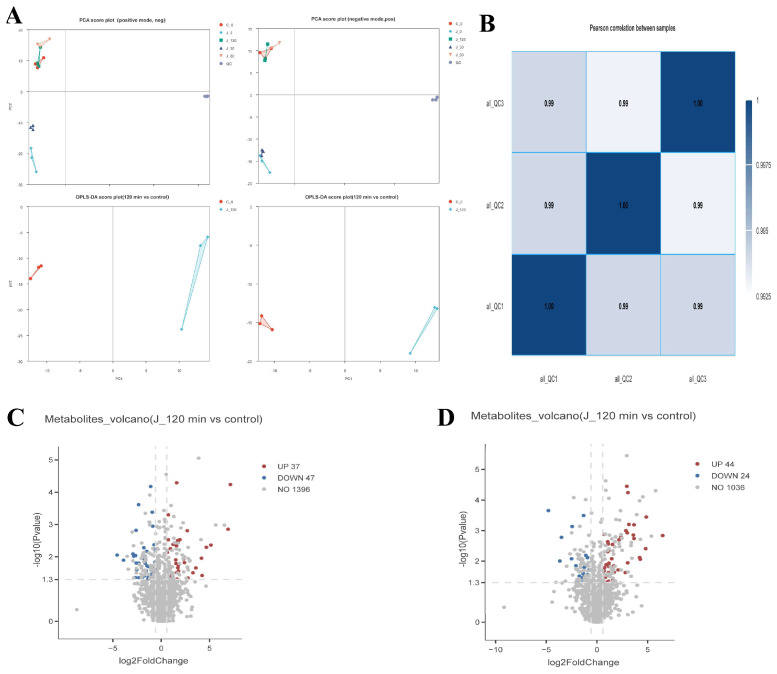
Multivariate statistical analysis plot of serum metabolomics in rats with JPAE intervention. (**A**) Multivariate statistical analysis including PCA score plots (positive/negative ion modes) and OPLS-DA score plots (J-120 min vs. control); (**B**) Pearson correlation heatmap among serum samples; (**C**) Volcano plot of differential metabolites in positive ion mode (J-120 min vs. control, UP: 37, DOWN: 47); (**D**) Volcano plot of differential metabolites in negative ion mode (J-120 min vs. control, UP: 44, DOWN: 24).

Multivariate statistical analysis revealed a clear separation trend among the control group (C_0), jujube-administered groups at different time points (J_0, J_30, J_60, J_120), and samples from each time point in the PCA score plots under both positive and negative ion modes (Figure 8). This separation suggested that JPAE intervention significantly altered the serum metabolic profiles of rats.

Under the current analytical conditions, the J_120 group exhibited the most pronounced separation from the control group (C_0) in the principal component space, whereas the separation of samples at other time points was relatively weak. These results indicated that the serum metabolic perturbation induced by JPAE intervention was most distinct at 120 min. Therefore, this time point was selected for subsequent differential metabolite identification and related biological mechanism analysis. To assess the stability of the detection platform and the reliability of the generated data, Pearson correlation analysis was performed on three quality control (QC) samples (all_QC1, all_QC2, all_QC3). The results revealed that the Pearson correlation coefficients between all pairs of QC samples were as high as 0.99, with the diagonal autocorrelation coefficients reaching 1.00.

#### 3.5.2. Cluster Analysis of Differential Serum Metabolites

**Figure 9:**
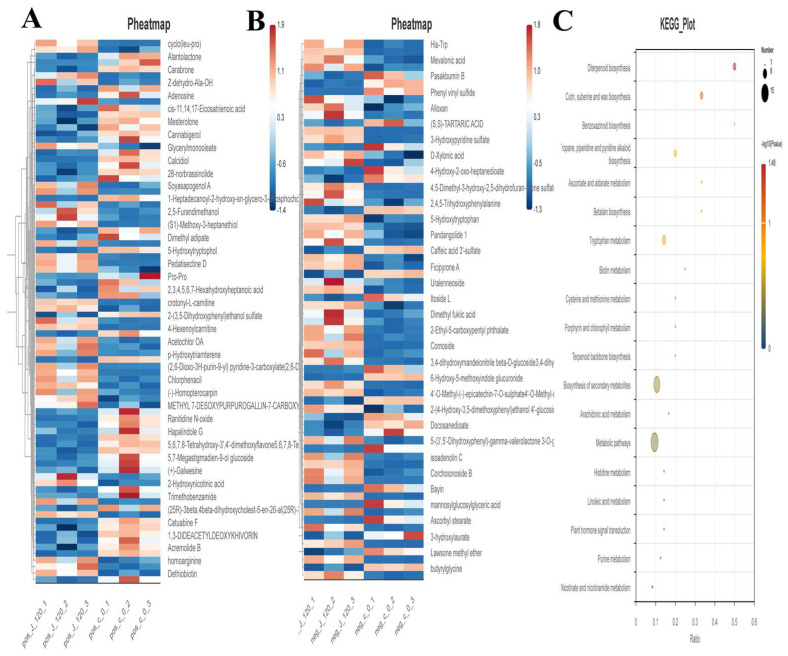
Cluster heatmap of differential serum metabolites and KEGG enrichment analysis plot of differential metabolites in rats with JPAE intervention. (**A**) Cluster heatmap of differential metabolites in positive ion mode; (**B**) Cluster heatmap of differential metabolites in negative ion mode; (**C**) KEGG pathway enrichment bubble plot of differential metabolites (bubble size represents gene count, color depth represents −log_10_ (*p*-value)).

The J_120 group (120 min after jujube gavage) and the blank control group (C group) were used as research objects, and differential metabolites were screened under positive and negative ion detection modes (screening criteria: *p* value ≤ 0.05, |log2FoldChange| ≥ 0.58, VIP ≥ 1). Under the positive ion mode (Figure 9A,B), 1480 metabolites were detected, including 37 significantly up-regulated metabolites, 47 significantly down-regulated metabolites, and 1396 metabolites with no significant difference. Under the negative ion mode, 1104 metabolites were detected, including 44 significantly up-regulated metabolites, 24 significantly down-regulated metabolites, and 1036 metabolites with no significant difference. Analysis was performed on differential serum metabolites between the jujube 120 min group and the control group. Phenolic acids underwent glucuronidation, sulfation [2], and O-methylation to form caffeic acid conjugates (caffeic acid 3′-sulfate), ferulic acid, etc., which were significantly up-regulated in the heatmap. Flavonoids underwent methylation and conjugation to form methylated catechins and quercetin conjugates, whose differential expression served as an important material basis for the hepatoprotective effect of flavonoids. Urolithins were produced from ellagic acid via metabolism by intestinal flora [26].

#### 3.5.3. Enrichment Analysis of Differential Metabolites

Enrichment analysis was conducted on the differential serum metabolites between the jujube 120 min administration group and the control group. KEGG pathway enrichment analysis showed (Figure 9) that the differential metabolites were mainly enriched in diterpenoid biosynthesis, biosynthesis of secondary metabolites, arachidonic acid metabolism, cysteine and methionine metabolism, and tryptophan metabolism pathways.

Literature studies have demonstrated that diterpenoids, as important active components in plants, exert anti-inflammatory and antioxidant activities following oral administration [27]. Among them, arachidonic acid metabolism was the core regulatory pathway of inflammatory responses [28,29,30], and its excessive activation was an important inducement of inflammatory [31]. Cysteine and methionine metabolism was directly associated with the synthesis of glutathione, the key antioxidant substance in vivo [32], which was closely associated with the pathological imbalance of oxidative stress, a key hallmark of tissue injury [33,34]. Tryptophan metabolism was a key target in the regulation of liver injury via the gut-liver axis [35], and its metabolic disorder was closely correlated with liver inflammation and impaired intestinal barrier function [36,37]. Previous studies have reported that flavonoids and phenolic acids in jujube mainly exist in the form of glycoside-bound precursors [38]. After oral administration, these components are metabolized and hydrolyzed in vivo to release active monomers, which is consistent with the differential metabolites detected in serum and the enrichment results of the biosynthesis pathway of secondary metabolites. These results were derived from serum metabolomics and pathway enrichment analysis, and only represent predictive correlations. The specific regulatory mechanisms of jujube active components on the above pathways and the pathological process of ALD still require further experimental verification.

By integrating serum metabolomics, network pharmacology, and molecular docking, this study comprehensively explored the metabolic regulatory mechanisms and key component targets of JPAE in physiological states. The in vivo effects of JPAE depend on its active constituents absorbed into the blood. As the direct material basis for physiological functions, these blood-available components served as a breakthrough point, overcoming the limitations of traditional studies that only focused on in vitro components [39]. Further analysis demonstrated that JPAE significantly remodeled the serum metabolic profile of healthy rats and regulated key pathways involved in lipid metabolism and secondary metabolite biosynthesis. These findings preliminarily clarified the holistic and multi-pathway regulatory characteristics of JPAE at the metabolic level, and also provided new insights for research on food-derived ingredients.

## 4. Conclusions

This study integrated serum metabolomics, network pharmacology, and molecular docking to systematically investigate the serum metabolic profiles and putative molecular mechanisms of JPAE. Quercetin, (-)-epigallocatechin, and methyl gallate were identified as prototype components in serum. These candidate absorbed compounds were further analyzed by network pharmacology and molecular docking. Serum metabolomic analysis revealed that the serum metabolic profiles were significantly remodeled at different time points after JPAE administration, with relatively marked metabolic changes observed at 120 min. Differentially accumulated metabolites were mainly enriched in pathways including metabolic pathways, diterpenoid biosynthesis, arachidonic acid metabolism, and biosynthesis of secondary metabolites, suggesting that JPAE may induce potential systemic metabolic modulation.

Nevertheless, this study has several limitations that should be acknowledged. First, the present study was carried out in healthy rats rather than an ALD model, which precluded the direct experimental validation of the protective effects of JPAE against ALD and its underlying causal mechanisms in vivo. Second, only serum metabolomics was analyzed. The potential synergistic regulation among multiple organs and the role of the gut-liver axis need to be further explored. Future studies will use classic ALD animal models to verify the functional effects of the putative absorbed components identified in this study. Multi-omics combined with gut microbiota analysis will also be used to elucidate the holistic regulatory network of JPAE. These efforts will provide a more reliable scientific basis for the further study and application of jujube and its related products.

## Figures and Tables

**Table 1:** MRM Mass Spectrometry Parameters.

Q1 (Da)	Q3 (Da)	Dwell (Msec)	DP (Volt)	CE (Volt)	ID
182.8	124	100	−68	−26	Methyl gallate
182.8	167.9	100	−68	−21	Methyl gallate
182.8	167.9	100	−68	−21	Methyl gallate
288.9	244.9	100	−82	−21	(-)-Epigallocatechin
288.9	124.8	100	−82	−29	(-)-Epigallocatechin
288.9	108.9	100	−82	−39	(-)-Epigallocatechin
300.9	151.1	100	−80	−30	Quercetin
300.9	121.1	100	−80	−31	Quercetin
300.9	178.9	100	−80	−27	Quercetin

(Note: CUR: 40; TEM: 500; GS1: 35; GS2: 65; IS: −4500; CAD: 9; CXP: −12).

**Table 2:** Blood-Absorbed Components from Jujube.

No	Metabolite Name	Molecular Formula	Rt (min)	Adduct Typy	Observed m/z	Reference m/z	Mass Error (ppm)	Area
1	Caffeic acid	C_9_H_8_O_4_	0.542	[M + H]+	181.05	181.0497	1.66	1.84 × 10^8^
2	Ellagic acid	C_14_H_6_O_8_	4.77	[M − H]−	300.998	300.9989	−3	4.12 × 10^7^
3	Quercetin	C_15_H_10_O_7_	5.867	[M + H]+	303.05	303.0501	−0.33	3.49 × 10^7^
4	(-)-Epicatechin	C_15_H_14_O_6_	5.762	[M + H]+	291.086	291.0863	−1.03	3.59 × 10^7^
5	Methyl gallate	C_8_H_8_O_5_	7.199	[M + H]+	185.04	185.04	0	1.01 × 10^9^
6	(-)-Epigallocatechin	C_15_H_14_O_7_	5.491	[M + H]+	307.081	307.0817	−2.28	3.65 × 10^8^
7	Ferulic acid	C_10_H_10_O_4_	5	[M − H]−	193.051	193.0506	2.07	6.14 × 10^8^
8	Chlorogenic acid	C_16_H_18_O_9_	2.419	[M + Na]+	377.08	377.0801	−0.27	3.02 × 10^7^
9	Arbutin	C_12_H_16_O_7_	5.229	[M + H]+	317.088	317.0868	3.79	7.43 × 10^7^
10	Gallic acid	C_7_H_6_O_5_	4.988	[M + CH_3_COO]−	331.065	331.0666	−4.83	4.72 × 10^7^

## Data Availability

The original contributions presented in the study are included in the article. The metabolomics data in this study have been registered in the Metabolomics Workbench with DataTrack ID: 7335 (mwTab file: mengyuan_liu_20260331_065421). All data will be publicly available upon acceptance of the manuscript.

## Abbreviations

JPAE	jujube paste aqueous extract
UPLC-MS/MS	Ultra Performance Liquid Chromatography–Tandem Mass Spectrometry
TCMSP	Traditional Chinese Medicine Systems Pharmacology Database and Analysis Platform
TTD	Therapeutic Targets Database
OMIM	Online Mendelian Inheritance in Man
KEGG	Kyoto Encyclopedia of Genes and Genomes
GO	Gene Ontology
LC-MS	Liquid chromatography-mass spectrometry
OPLS-DA	Orthogonal partial least squares discriminant analysis
PCA	Principal component analysis
SD	Standard deviation
VIP	Variable importance in projection
QC	quality control
BPCs	base peak chromatogram
AKT1	serine/threonine kinase 1
EGFR	Epidermal growth factor receptor
HSP90AA1	Heat shock protein 90 alpha family class A member 1
JAK2	Janus kinase 2
MAPK1	Mitogen-activated protein kinase 1
PIK3R1	Phosphoinositide-3-kinase regulatory subunit 1
PTK2	Protein tyrosine kinase 2
SRC	SRC proto-oncogene, non-receptor tyrosine kinase
